# The Effects of Supplementation with a Vitamin and Mineral Complex with Guaraná Prior to Fasted Exercise on Affect, Exertion, Cognitive Performance, and Substrate Metabolism: A Randomized Controlled Trial

**DOI:** 10.3390/nu7085272

**Published:** 2015-07-27

**Authors:** Rachel C. Veasey, Crystal F. Haskell-Ramsay, David O. Kennedy, Karl Wishart, Silvia Maggini, Caspar J. Fuchs, Emma J. Stevenson

**Affiliations:** 1 Brain, Performance and Nutrition Research Centre, Faculty of Health and Life Sciences, Northumbria University, Newcastle upon Tyne NE1 8ST, UK; E-Mails: crystal.haskell-ramsay@northumbria.ac.uk (C.F.H.-R.); david.kennedy@northumbria.ac.uk (D.O.K.); cas.fuchs@northumbria.ac.uk (C.J.F.); e.stevenson@northumbria.ac.uk (E.J.S.); 2 Bayer Consumer Care AG, Peter Merian Strasse 84, P.O. Box, Basel 4002, Switzerland; E-Mails: karl.wishart@bayer.com (K.W.); silvia.maggini@bayer.com (S.M.)

**Keywords:** micronutrients, guaraná, exercise, cognition, memory, mood

## Abstract

Exercise undertaken in a fasted state can lead to higher post-exercise mental fatigue. The administration of a vitamin and mineral complex with guaraná (MVM + G) has been shown to attenuate mental fatigue and improve performance during cognitively demanding tasks. This placebo-controlled, double-blind, randomized, balanced cross-over study examined the effect of MVM + G consumed prior to morning exercise on cognitive performance, affect, exertion, and substrate metabolism. Forty active males (age 21.4 ± 3.0 year; body mass index (BMI) 24.0 ± 2.4 kg/m^2^; maximal oxygen consumption (V̇O_2max_) 57.6 ± 7.3 mL/min/kg) completed two main trials, consuming either MVM + G or placebo prior to a 30-min run at 60% V̇O_2max_. Supplementation prior to exercise led to a small but significant reduction in Rating of Perceived Exertion (RPE) during exercise compared to the placebo. The MVM + G combination also led to significantly increased accuracy of numeric working memory and increased speed of picture recognition, compared to the placebo. There were no significant effects of supplementation on any other cognitive or mood measures or on substrate metabolism during exercise. These findings demonstrate that consuming a vitamin and mineral complex containing guaraná, prior to exercise, can positively impact subsequent memory performance and reduce perceived exertion during a moderate-intensity run in active males.

## 1. Introduction

Many active individuals exercise in a fasted state before breakfast for reasons including time constraints and to avoid discomfort during exercise e.g., [1]. They may also choose to exercise in a fasted state due to the associated increase in fat oxidation compared to exercise following food, a hypothesis proven in previous laboratory studies [2,3]. Although nutritional guidelines are available regarding improvement to exercise performance [4], relatively little is known about how pre-exercise nutrition affects post-exercise cognitive performance and mood state. However, a recent study demonstrated that exercising in a fasted, compared to fed, state leads to higher post-exercise mental fatigue [5].

The observed impact of fasting on post-exercise mental fatigue potentially relates to the micronutrient status of participants. Restriction of energy intake or omission of specific food groups may put those who are physically active at increased risk of experiencing the marginal deficiency of one or more vitamins or minerals observed in the general population of developed countries [6,7,8]. In addition, biochemical changes and heightened metabolic demands that occur during exercise lead to increases in the requirements for certain micronutrients. This is further exacerbated by higher excretion of micronutrients through waste products, such as sweat and urine, during and after strenuous exercise [9]. Micronutrients are involved in a multitude of processes important to physical and mental performance. Of relevance to the current investigation, B vitamins are necessary for the metabolism of carbohydrates, amino acids, and fat, and serve as co-factors in many key reactions in energy-producing pathways, including mitochondrial electron transfer. They are also essential for the regeneration of cells such as red blood cells and are involved in DNA synthesis and maintenance of the myelin sheath (for reviews see [10,11]). B vitamins are also inversely correlated with the potentially neurotoxic amino acid homocysteine, which in turn is positively correlated with cardiovascular disease, age-associated cognitive decline, and dementia [12]. Evidence also suggests that vitamin C contributes to tissue repair and provides a range of functions within the central nervous system , including contributing to the synthesis of neurotransmitters and direct binding to cognition relevant receptors throughout the brain [13]. The minerals zinc, magnesium, and calcium are also important due to their roles in cell metabolism, cardiovascular and hormonal functions, oxygen uptake, and nerve conduction [14,15,16]. Given the important functions of micronutrients, it is likely that those who are physically active, and consume an inadequate diet, might benefit from supplementation to prevent impairment to physical and psychological outcomes.

Previous research has indicated that chronic supplementation with a multivitamin mineral complex (MVM), with and without additional herbal extracts, can improve performance of behavioral tasks and increase measures of alertness in young and older adult males and females [17,18,19,20]. As a consequence of MVM supplementation, reductions in homocysteine have also been observed [17,19,20], levels of which have been shown to both increase and decrease as a consequence of exercise [21,22,23].

Guaraná, an extract made from the berries of the Amazonian plant *Paullinia cupana*, is an increasingly common component in energy drinks that has previously been shown to improve measures of attention and secondary memory, and to increase alertness and contentment ratings [24,25]. Its stimulant properties are usually attributed to its caffeine content (2.5–5% of dry weight), which is known to modify cognitive function and mood state [26] and to enhance exercise performance, possibly due to a reduction in perceived exertion [27]. Caffeine consumed in a beverage is absorbed by the small intestine within 45 min of ingestion [28], and previous data show no difference in absorption rates between pure caffeine and caffeine from guaraná [29]. However, guaraná also contains the methylxanthines theophylline and theobromine [30], as well as saponins and polyphenols, including tannins, catechins, and epicatechins [31], and differences in the behavioral effects of caffeine and guaraná have been noted [32]. In addition, a recent meta-analysis reported that although both caffeine and tea catechin-caffeine mixtures increased energy expenditure, increases to fat oxidation only reached significance following the mixture [33]. Fat oxidation increases of a similar magnitude have been shown following calcium, and MVM supplementation has been shown to affect metabolism both chronically [34] and acutely [35]. These data raise the possibility that combining MVM with guaraná (MVM + G) may produce synergistic effects. Support for this comes from a recent study directly comparing the acute effects of high-dose B-complex MVM (Berocca Performance^®^, Bayer, Leverkusen, Germany) with a slightly lower dose B-complex MVM + G (Berocca Boost^®^, Bayer), which demonstrated greater brain activation (in the left and right superior parietal lobe and the right cerebellum, as assessed by functional magnetic resonance imaging following MVM + G than MVM as well as behavioral improvements only seen with MVM + G [36]. Of particular relevance to the current study, MVM + G resulted in improvements to speed and accuracy of a rapid visual information processing task (RVIP) when measured during a 10-min cognitive demand battery (CDB), beginning 30 min post-supplementation. Earlier data also show an increase in mental fatigue associated with completion of the CDB was attenuated by supplementation with MVM + G [37].

The involvement of vitamins and minerals in energy-producing pathways and previous data showing the metabolic effects of both micronutrients and guaraná suggest that enhancement of metabolically challenging tasks may be possible. Indeed, evidence of improved cognitive task performance (attention, and working and secondary memory) and reduced mental tiredness/fatigue during cognitive demand following MVM, both with and without guaraná [18,24,25,37], supports this notion. The positive data on the effect of guaraná on alertness and contentment also advocates further testing of these constituents on subjective mood state, particularly measures related to mental fatigue such as stamina, tiredness, and perceived exertion. Exercise has been described as a metabolic stressor and as such provides an excellent paradigm to explore the potential synergistic effects of MVM + G. The present study, therefore, investigated the impact of a complex containing vitamins, minerals, and guaraná (Berocca Boost^®^) consumed pre-exercise on cognitive performance and mood state measured pre- and post-exercise, substrate metabolism during exercise, and affect measured during exercise and in the post-exercise recovery period. It was expected that MVM + G supplementation would reduce any cognitive and mood detriments created by the fasted exercise intervention.

## 2. Methods

### 2.1. Study Design

This study employed a randomized, double-blind, placebo-controlled, balanced cross-over design. The baseline measurements provided a means for controlling individual differences in mood and cognitive task performance on each study day. The study was conducted at a single research center (the Brain, Performance and Nutrition Research Centre, Northumbria University). Ethical approval (RE-HLS-12-130625-51c986ece082a; 18 July 2013) was obtained from Northumbria University Faculty of Health and Life Sciences Ethics Committee and the study was conducted according to the Declaration of Helsinki (2013).

### 2.2. Participants

Participants were recruited via email distribution to both students and staff at the university. Following the screening and informed consent process (*n* = 45), 43 participants were enrolled in the study. Three participants withdrew consent before randomization, leaving a final sample of 40 active males who completed the study.

All participants reported to be male, in good health, and free from prescription medication and herbal extracts/food supplements. Participants who had suffered a head injury or a neurological, psychiatric (including depression and anxiety), metabolic, endocrine, or cardiac disorder were excluded from participation, as were those who had any relevant food allergies or intolerances, smoked tobacco, had a history of drug or alcohol abuse, drank excessive amounts of caffeine (>500 mg/day), or had a high blood pressure (>140/90 mmHg) or body mass index (BMI; above 30 kg/m^2^). Participants also confirmed that they were regularly active (exercising at least twice per week) and able to run non-stop on a treadmill at a moderate pace for 30 min.

### 2.3. Study Interventions

Each participant received both the multivitamin and mineral complex with guaraná (Berocca Boost^®^, hereafter referred to as MVM + G) and placebo on separate days, at least seven days apart, in an order dictated by random allocation to a counterbalancing schedule. Computer-generated random numbers were used to allocate treatment order to participant identification numbers. On the test day, the treatment or placebo effervescent tablet was dissolved in 250 mL of water. The test drinks were equal in taste and appearance, but were served in an opaque beaker with a lid and consumed through a straw to be certain blindness to the treatment was maintained. The treatment tablet contents are detailed in Table 1.

**Table 1:** Contents of the treatment tablet (MVM + G).

Active	Units	Berocca Boost^®^	% of RDA *	European RDA *^†^	UL ^‡^
Vitamin B_1_	mg	1.4	127	1.1	nd
Vitamin B2	mg	1.6	114	1.4	nd
Nicotinamide (B3)	mg	18	113	16	35 ^a^
Pantothenic acid (B5)	mg	6	100	6	nd
Vitamin B6	mg	2	143	1.4	100 ^a^
Biotin	µg	150	300	50	nd
Folic acid (B9)	µg	200	100	200	1000 ^a^
Vitamin B12	µg	1	40	2.5	nd
Vitamin C	mg	60	75	80	2000 ^b^
Calcium	mg	100	12.5	800	2500 ^c^
Magnesium	mg	100	27	375	350 (suppl. only) ^c^
Zinc	mg	9.5	95	10	40 ^d^
Guaraná	mg	222.2 (containing 40 mg of caffeine)			-

* European recommended daily allowance; **^‡^** Tolerable upper intake level; **^†^** [38]; ^a^ [39]; ^b^ [40]; ^c^ [41]; ^d^ [42]; nd, not detected.

### 2.4. Cognitive and Mood Assessments

Cognitive and mood assessment was undertaken using COMPASS (Computerized Mental Performance Assessment System, University of Northumbria at Newcastle) and comprised a selection of standard tasks. COMPASS has been used in several previous nutritional intervention studies and has been shown to be sensitive to cognitive enhancement following supplementation of a variety of substances [18,43,44,45] including in combination with exercise [5]. The tasks chosen allowed an assessment focusing on secondary and working memory, attention, and decision-making, and were administered 30, 75, and 150 min post-dose. Both task choice and timing of task administration were selected based on data from previous guaraná [24,25] and MVM + G [36] studies. Each cognitive/mood assessment was identical, with the exception that unique, randomly organized stimuli were used on each occasion.

The selection of visual analogue scales (VAS), stimuli presentations and tasks comprised:

#### 2.4.1. Bond-Lader VAS

Sixteen scales (100 mm lines) labeled at either end by antonyms (e.g., “alert-drowsy”, “calm-excited”) were presented. Participants indicated their current subjective position between the antonyms on the line. Outcomes comprised three factor analysis derived scores: “Alertness”, “Calmness” and “Contentment” [46]. These scales were originally designed for assessing the mood effects of anxiolytics and have been subsequently utilized in numerous pharmacological, psychopharmacological, and medical trials. Visual analogue scales have been shown to have high reliability and validity for the measurement of mood [47] and a previous data suggests an improvement in contentment and alertness following guaraná administration when measured using the Bond-Lader VAS [24].

#### 2.4.2. Energy VAS (Concentration, Physical Stamina, Mental Stamina)

Participants completed three VAS labeled at either end by “very low” and “very high” measuring levels of “concentration”, “mental stamina” and “physical stamina”.

Data for all VAS comprised scores in mm along the line from left to right.

#### 2.4.3. Picture Presentation (~1 min)

Twenty pictures of everyday objects appeared on the screen, one at a time. One picture was presented every 2 s, with a stimulus duration of 1 s. Participants were required to look carefully at these pictures and try to remember the details in them.

#### 2.4.4. Word Presentation and Immediate Word Recall (~2 min)

Fifteen words appeared in the middle of the screen, one at a time. Stimulus and inter-stimulus duration was 1 s. Participants were instructed to try and remember as many as they could. Immediately afterwards, they had one minute to write down as many of the words as they could remember.

#### 2.4.5. Choice Reaction Time (CRT, ~2 min)

An arrow pointing LEFT or RIGHT appeared on the screen at irregular intervals. Participants were required to press the corresponding button on the response pad, as quickly and as accurately as they could, following the presentation of an arrow.

#### 2.4.6. Rapid Visual Information Processing Task (RVIP, 5 min)

A series of single digit numbers between 1 and 9 appeared on the screen continuously at a rate of 100 per·min^−1^. Participants pressed the center button on the response box, reacting as quickly and accurately as possible, when they identified three odd or even digits presented in succession.

#### 2.4.7. Numeric Working Memory (NWM, 3 min)

Five numbers appeared on the screen, one at a time. Participants were asked to remember these five numbers. A series of numbers then appear on the screen, one at a time. For each of these numbers, participants identified whether the number presented was one from the original five they were shown. This was repeated three times in total, with a new set of five numbers presented for each completion.

#### 2.4.8. Delayed Word Recall (1 min)

Participants were asked to write down as many of the words they could remember from the previous word presentation task.

#### 2.4.9. Word Recognition (~1 min)

Participants were shown 15 target words and 15 distractor words on the screen, presented one at a time. Participants identified whether the word shown was shown in the original word presentation task or not.

#### 2.4.10. Picture Recognition (~1 min)

Participants were shown a series of 10 target pictures and 10 distractor pictures on the screen, presented one at a time. Participants identified whether the picture shown was shown in the original picture presentation task or not.

Data from the cognitive tasks comprised mean speed (ms) and % accuracy scores for each task except the Immediate and Delayed Word Recall tasks, which were scored for number of correct responses and errors.

### 2.5. Additional Assessments

#### 2.5.1. Felt Arousal Scale (FAS) and Feeling Scale (FS)

Participants were asked to rate their level of arousal using the FAS [48] and FS [49] immediately before and after exercise and at 10 min intervals during the exercise period. Further ratings were recorded at 30 and 60 min post-exercise. Data from the FAS comprised scores between 1 (low arousal) and 6 (high arousal), FS data comprised scores between −5 (very bad) and +5 (very good). The FAS and FS are widely used to assess affect in an exercise setting.

#### 2.5.2. Rating of Perceived Exertion (RPE)

RPE [50] was rated at 10 min intervals during exercise using the Borg Category 20 scale. RPE data comprised scores between 6 (no exertion at all) and 20 (maximal exertion).

#### 2.5.3. Indirect Calorimetry (ICa)

Expired air samples were collected via an online gas analysis system (Metalyzer 3B, Cortex, Leipzig, Germany) at 9, 19, and 29 min during the exercise bout. Participants wore the mask 2.5 min before each sampling point and then a 60 s collection time commenced. Respiratory exchange ratio (RER), oxygen uptake, and carbon dioxide production were measured at each sampling point in order to calculate fat and carbohydrate oxidation and energy expenditure. Substrate metabolism was calculated, assuming minor protein oxidation, with V̇O_2_ and CO_2_ production values using stoichiometric equations adjusted during exercise to account for the contribution of glycogen to metabolism [51].

### 2.6. Study Procedure

#### 2.6.1. Visit 1 (Screening and Training)

All participants gave voluntary written informed consent prior to their inclusion in the study. They then completed a screening session to confirm their eligibility to take part including a health questionnaire to highlight any potential issues of their participation in a trial involving exercise and supplementation. Suitable participants underwent training on the computerized cognitive tasks, which were repeated three times during this session to decrease the chance of learning effects during main trials. Participants also completed two preliminary tests to determine: (1) the relationship between maximal oxygen consumption (V̇O_2max_) and running speed on a flat treadmill using a 16-min test and (2) their V̇O_2max_ using an incremental treadmill test whereby the gradient was increased by 1%·min^−1^ to exhaustion as previously described [52].

#### 2.6.2. Visit 2 (Exercise Familiarization)

Participants visited the research center at least 48 h after Visit 1 to complete a familiarization run. This provided confirmation, and opportunity for adjustment of the running speed calculated to be 60% V̇O_2max_ (using data collected during the preliminary tests at visit 1). If average V̇O_2_ was >2.5% above or below that calculated to be 60%, speed adjustments were then made at the discretion of the researcher. It has been suggested the effect of caffeine on fat oxidation may only become apparent at lower exercise intensities than the high intensity exercise interventions used in studies to date [53] and 30 min at this moderate level reflects a typical exercise session undertaken by habitually active individuals. In addition, moderate-intensity fasted exercise has been shown to increase mental fatigue [5].

#### 2.6.3. Visit 3 (Main Trial 1)

Participants visited the laboratory at least 48 h after Visit 2 to complete the first Main Trial. For 24 h before this visit, participants recorded their food intake and avoided alcohol, strenuous exercise, and intake of non-prescription medications. On the morning of the trial, participants reported to the laboratory following a minimum of 10 h overnight fast (no food or drink other than water). Before commencement of the trial, continued conformity with the inclusion and exclusion criteria, alcohol use (confirmed using an alcohol breath test), concomitant medication and dietary supplement use, and adverse events since the last visit were reviewed.

**Figure 1:**
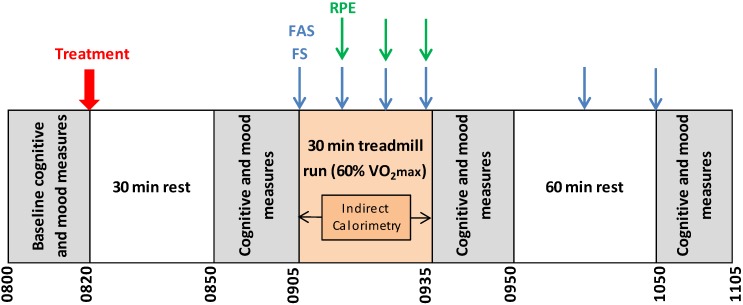
Overview of study trial days (Visit 3 and 4).

Following baseline cognitive and mood measurements, participants were provided with the intervention and then rested for 30 min in the laboratory. After 30 min, cognitive and mood measures were repeated before participants completed a 30-min run at 60% V̇O_2max_. This was immediately followed by completion of the cognitive tasks and mood assessments. Participants then rested in the laboratory for the following 60 min before completing a final assessment of cognitive performance and mood. ICa was measured continuously throughout the exercise period. RPE, FAS, and FS ratings were recorded at 10 min intervals throughout exercise and FAS and FS at 45 and 75 min post-exercise also. Each battery of cognitive tasks and mood scales took ~18 min to complete. During rest periods, participants remained in the laboratory and watched a video provided by the researcher. Water intake was recorded during Visit 3 and consumption of the same amount was encouraged at Visit 4. An overview of the study trial days is presented in Figure 1.

#### 2.6.4. Visit 4 (Main Trial 2)

Visit 4 was identical to Visit 3, with the exception of the drink administered, and took place at least seven days following Visit 3. Participants were asked to consume the same foods in the 24 h period prior to Visit 4 as recorded in the food diary. After completion of all trial procedures, participants were fully debriefed.

### 2.7. Statistical Analysis

Before the main statistical analyses, paired sample *t*-tests were utilized to compare pre-dose baseline data (where applicable) to adjust for differences in performance across the study days. Scores for the cognitive task and mood outcomes were then analyzed as change from baseline using a 2 × 3 (treatment × time) repeated measures analysis of variance (ANOVA). Scores for the FS and FAS data were analyzed as absolute values using a 2 × 6 (treatment × time) repeated measures ANOVA and RPE and substrate metabolism as absolute values using a 2 × 3 (treatment × time) repeated measures ANOVA. To correct for multiple comparisons, the Bonferroni correction was applied. An alpha level of 0.05 was used for all statistical tests. Mean scores and ratings are reported with the Standard Deviation (SD). Effect sizes for significant results are reported using Cohen’s D (*d*).

## 3. Results

The characteristics of the final sample are detailed in Table 2. The mean V̇O_2__max_ of 57.6 ± 7.3 mL/min/kg indicates that the sample was highly trained.

**Table 2:** Participant characteristics.

Characteristic	Mean ± SD
Age (years)	21.4 ± 3.0
Weight (kg)	77.3 ± 9.1
Height (m)	1.8 ± 0.1
BMI (kg/m^2^)	24.0 ± 2.4
V̇O_2__max_ (mL/min/kg)	57.6 ± 7.3
Habitual caffeine intake (mg/day)	77.7 ± 82.3

SD: standard deviation.

### 3.1. Baseline Differences

No significant baseline differences were found for any outcome. See Tables S1–S4, which show change from baseline or absolute values for all measures (as appropriate) and significant effects of time.

### 3.2. Study Outcome Variables

No treatment x time interactions were found for any of the variables. No significant main effects of treatment were found for the FAS, FS, energy VAS, Bond-Lader mood scales, substrate metabolism, RER, or energy expenditure (EE, see Tables S2–S4). Significant main effects of treatment for cognitive performance and ratings of perceived exertion are presented below.

### 3.3. ICa (See Table S4)

There were no effects of treatment on any ICa measure.

### 3.4. Cognitive Performance (See Table S1)

No significant effects of treatment were found for CRT, RVIP, NWM reaction time (RT), Word Recall and Recognition, or Picture Recognition accuracy.

#### 3.4.1. Numeric Working Memory (NWM)

**Figure 2:**
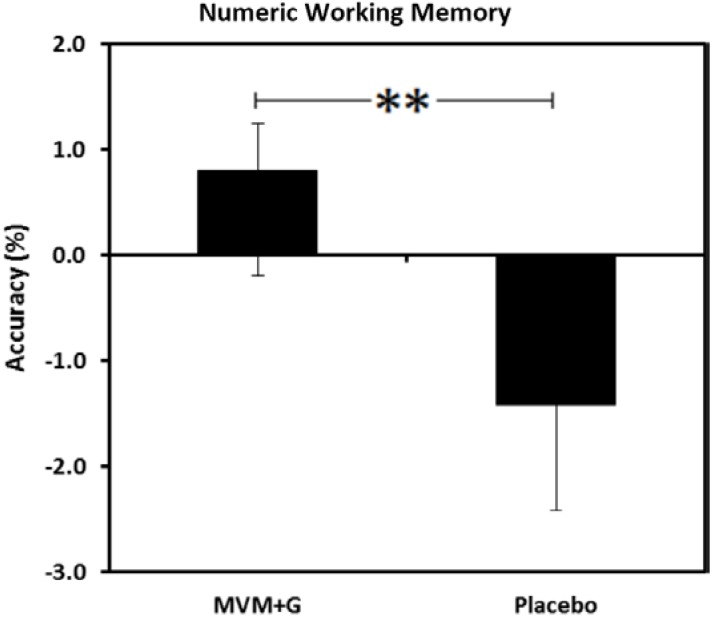
Mean accuracy scores (%) on a numeric working memory task following consumption of a multi-vitamin and mineral complex with guaraná (MVM + G) or placebo prior to exercise. Values are change from baseline, ** *p* < 0.01.

A main effect of treatment was found for NWM accuracy (F(1,35) = 14.10, *p* = 0.001, *d* = 0.71), with better accuracy seen following consumption of MVM + G compared with the placebo (Figure 2).

#### 3.4.2. Picture Recognition

**Figure 3:**
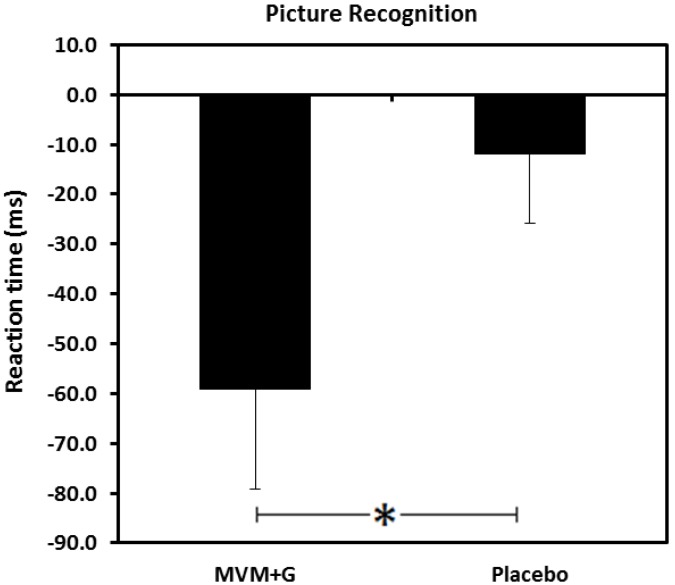
Mean reaction times (ms) on a picture recognition task following consumption of a multi-vitamin and mineral complex with guaraná (MVM + G) or placebo prior to exercise. Values are change from baseline, * *p* < 0.05.

A main effect of treatment was found for Picture Recognition RT (F(1,37) = 4.12, *p* = 0.0496, *d* = 0.40), with a faster RT seen following consumption of MVM + G compared with the placebo (Figure 3).

### 3.5. Rating of Perceived Exertion (RPE) (See Table S3)

**Figure 4:**
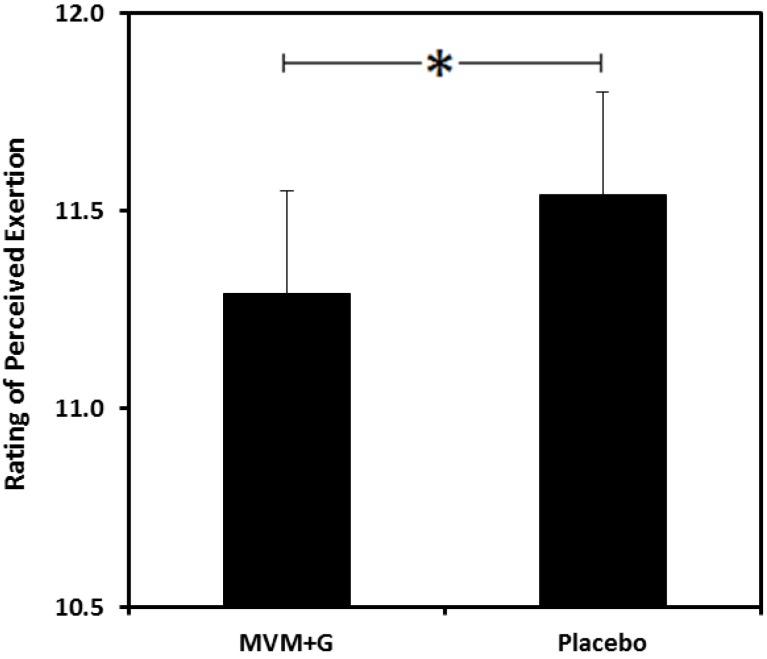
Mean rating of perceived exertion during exercise following prior consumption of a multi-vitamin and mineral complex with guaraná (MVM + G) or placebo. Values are absolute, * *p* < 0.05.

A main effect of treatment was found for RPE during exercise (F(1,39) = 5.57, *p* = 0.023, *d* = 0.14), where RPE was lower following consumption of MVM + G compared with the placebo (Figure 4).

### 3.6. Mood and Affect (See Table S2)

There were no effects of treatment on any measure of mood or affect.

### 3.7. ICa (See Table S4)

There were no effects of treatment on any ICa measure.

## 4. Discussion

Findings from the present study demonstrate that a multi-vitamin and mineral complex with guaraná (MVM + G) consumed prior to a bout of moderate-intensity exercise can attenuate the rise in perceived exertion during exercise and augment memory performance (working memory accuracy and speed of episodic memory), measured before and after exercise, in active males.

Previously, consumption of a MVM + G, identical to the one administered in the current study, has been shown to improve cognition and attenuate mental fatigue produced during a cognitive demand paradigm [37]. The results from the current study showed that whilst accuracy on the numeric working memory task declined with the placebo, it improved following MVM + G. A decrease in reaction time on the picture recognition reaction task was seen following both interventions but this was significantly augmented by MVM + G. As with the majority of computerized cognitive tasks, numeric working memory and picture recognition involve aspects of attention and psychomotor response, but the predominant cognitive domain utilized during these tasks is memory (working and long term memory, respectively). This supports previous evidence of increased brain activation associated with working memory and attention when measured following MVM and MVM + G. These effects were amplified with the complex containing guaraná, which also improved performance on a serial threes task and increased contentment, suggesting a beneficial synergy between these constituents [36].

The positive cognitive results seen in the current study were present in the absence of a speed/accuracy trade-off, although it should be noted that memory augmentation occurred in only two of the five memory tasks, and that a consistent effect on accuracy or speed, or type of memory affected was not found. We were also unable to replicate a previously demonstrated improvement to the RVIP task following MVM + G [37]. This is potentially due to the lower number of repetitions completed in the current study, a proposition supported by data from [36], also showing no effect of MVM + G on this task when only completed once. This suggests that the effects of this supplement may be more pronounced when task demands are high and/or mental fatigue has been induced, at least more so than in the current study; more challenging tasks may also reduce the possibility of ceiling effects. We proposed that fasted exercise may act as a metabolic stressor and leave individuals vulnerable to cognitive detriments due to a reduction in available micronutrients, which are involved in a plethora of cognitive processes, and that, in this state, MVM + G supplementation would be beneficial As moderate-intensity fasted exercise has been shown to increase mental fatigue [5], the current study adopted a similar method to induce a fatigued state. However, the positive effects of supplementation observed on cognition were apparent irrespective of whether they were measured pre- or post-exercise, indicating that these effects emerge as early as 30 min post-consumption and are unaffected by the exercise bout employed here. Reference to effects over time indicates that the exercise session resulted in increased alertness immediately post-exercise and felt arousal ratings also increased as a result of exercise, with highest ratings immediately after exercise. This suggests that the exercise bout employed here had a stimulating rather than fatiguing effect, which supports a recent review indicating that acute exercise enhances feelings of energy [54]; it may be more likely that a state of fatigue would be induced if high, rather than moderate, intensity exercise was performed [55,56]. This failure of the exercise paradigm to induce fatigue may explain the lack of interactive effects but it should be considered that a 30-min bout of moderate intensity exercise is likely reflective of that which is regularly undertaken by the general population. Interactive effects may also have been more likely to be detected if measurements had been taken later in the day, as suggested by significantly lower choice reaction accuracy, arousal, and alertness ratings when measured 75 min post-exercise, compared to pre-exercise.

A reduction in perceived exertion during exercise was also observed following supplementation. An obvious constituent of the treatment provided in the current study, which may have delivered this effect, is caffeine. Caffeine stimulates the central nervous system [26,57] and a previous meta-analysis concluded that caffeine consumption can reduce perceived exertion during exercise [58]. The enhancing properties of caffeine during exercise can be attributed to its ability to block and inhibit adenosine receptors [57,59]. This action stimulates the release of adrenaline, which can reduce breathing effort during exercise through an increase in respiratory muscle endurance [60] and a subsequent increase in ventilation [61]. In the current study, based on the mean body weight of the participants, 40 mg caffeine equated to just 0.52 mg/kg. In the studies included in Doherty and Smith’s analysis, the lowest amount of caffeine administered prior to exercise was 4 mg/kg of body weight. Although the minimum effective dose of caffeine for improvements in RPE has not been established [62], it is perhaps unlikely that caffeine was solely responsible for the effect seen in the current study due to the small dose provided. In addition, it should also be considered that caffeine administered via guaraná may have different influences than when administered in isolation if the other constituents of this compound (some of which may have psychoactive properties, such as saponins and tannins [31]) act synergistically with the caffeine [24,63]. However, there has been very limited research investigating the effect of guaraná only, consumed prior to exercise, in human subjects, and no known studies to date have directly compared this compound with caffeine in this scenario; the inclusion of a caffeine-only condition would have been desirable for this reason and is acknowledged as a limitation in the current study. When we consider that a multi-vitamin/mineral complex without guaraná has previously been shown to increase vigor [18] and physical stamina [64] following a 33-day intervention study, it is possible that both the vitamin/mineral and guaraná components of the supplement may have contributed to the effect we see on perceived exertion. No study to date has examined whether acute B-vitamin supplementation prior to exercise can reduce perceived exertion during exercise, but our data provide some support for this and there are a multitude of mechanisms to suggest that it would. B vitamins are essential for macronutrient and amino acid metabolism and serve as co-factors in many key reactions in energy-producing pathways (for reviews see [10,11]).

Recreationally active individuals may be more likely to exercise in a fasted state due to the associated increase in fat oxidation [2,3]. Some data have shown that consuming caffeine prior to fasted exercise can enhance this effect [65], although this theory is not well supported. It has been suggested that the effect of caffeine on fat oxidation may only become more apparent during moderate intensity exercise, as implemented in the current study, which is lower than that used in most studies to date [53]. However, our data do not support this theory. It could be that an intensity of 60%·V̇O_2max_ is still too high to observe these effects of caffeine or that the relatively low dose administered is ineffective. It would of course be interesting to observe the effect of this supplement when consumed prior to exercise of various intensities. Interestingly, augmented fat oxidation at rest has been reported following acute consumption of a MVM complex without guaraná [35]. Although fat metabolism during exercise did not differ significantly between the conditions in the current study, it could be suggested that pre-exercise ingestion of MVM + G may be beneficial in order to maintain higher fat oxidation (when compared to the effect of consuming macronutrients prior to exercise [2,3]) and make exercise feel less strenuous.

## 5. Conclusions

In summary, MVM + G consumed 1 h prior to moderate-intensity exercise can reduce feelings of exertion during exercise and improve cognitive performance up to 90 min post-exercise. Active individuals who wish to fast prior to exercise may benefit from the consumption of this product pre-exercise to make moderate-intensity exercise feel easier, maintain a higher fat oxidation compared to the consumption of macronutrients, and alleviate any possible negative cognitive effects post-exercise. Currently, this data only extends to an active male population; as active females generally have poorer nutrient intake than males [66], it is important that these effects are explored in women. Future research should also be directed at exploring the impact of altering exercise intensity and how these effects differ over the course of the day. The plethora of mechanisms pointing toward effects of multivitamins/minerals on energy expenditure and recent evidence for a significant acute effect of supplementation of this type on metabolism [35], suggest that the impact of MVM minus guaraná warrants further research following both acute and chronic supplementation.

## Supplementary Files

Supplementary File 1

## References

[1] Hill K.M., Whitehead J.R., Goodwin J.K. (2011). Pre-workout carbohydrate supplementation does not affect measures of self-assesed vitality and affect in college swimmers. J. Sports Sci. Med..

[2] Backhouse S.H., Williams C., Stevenson E., Nute M. (2007). Effects of the glycemic index of breakfast on metabolic responses to brisk walking in females. Eur. J. Clin. Nutr..

[3] Gonzalez J.T., Veasey R.C., Rumbold P.L.S., Stevenson E.J. (2013). Breakfast and exercise contingently affect postprandial metabolism and energy balance in physically active males. Br. J. Nutr..

[4] Hargreaves M., Hawley J.A., Jeukendrup A. (2004). Pre-exercise carbohydrate and fat ingestion: Effects on metabolism and performance. J. Sports Sci..

[5] Veasey R.C., Gonzalez J.T., Kennedy D.O., Haskell C.F., Stevenson E.J. (2013). Breakfast consumption and exercise interact to affect cognitive performance and mood later in the day. A randomized controlled trial. Appetite.

[6] Adams J.S., Hewison M. (2010). Update in vitamin D. J. Clin. Endocrinol. Metab..

[7] Ruston D., Hoare J., Henderson L., Gregory J., Bates C.J., Prentice A., Birch M., Swan G., Farron M. (2004). Volume 4: Nutritional status (anthropometry and blood analytes), blood pressure and physical activity. The National Diet and Nutrition Survey: Adults Aged 19 to 64 Years.

[8] Schleicher R.L., Carroll M.D., Ford E.S., Lacher D.A. (2009). Serum vitamin C and the prevalence of vitamin C deficiency in the United States: 2003–2004 National Health and Nutrition Examination Survey (NHANES). Am. J. Clin. Nutr..

[9] Manore M.M. (2000). Effect of physical activity on thiamine, riboflavin, and vitamin B-6 requirements. Am. J. Clin. Nutr..

[10] Maughan R.J. (1999). Role of micronutrients in sport and physical activity. Br. Med. Bull..

[11] Woolf K., Manore M.M. (2006). B-vitamins and exercise: Does exercise alter requirements?. Int. J. Sport Nutr. Exerc. Metab..

[12] Kennedy D.O., Haskell C.F. (2011). Vitamins and cognition. Drugs.

[13] Harrison F.E., May J.M. (2009). Vitamin C function in the brain: Vital role of the ascorbate transporter SVCT2. Free Radic. Biol. Med..

[14] Lukaski H.C. (2004). Vitamin and mineral status: Effects on physical performance. Nutrition.

[15] Nielsen F.H., Lukaski H.C. (2006). Update on the relationship between magnesium and exercise. Magnes. Res..

[16] Rodriguez N.R., DiMarco N.M., Langley S. (2009). Nutrition and athletic performance. Med. Sci. Sports Exerc..

[17] Haskell C.F., Robertson B., Jones E., Forster J., Jones R., Wilde A., Kennedy D.O. (2010). Effects of a multi-vitamin/mineral supplement on cognitive function and fatigue during extended multi-tasking. Hum. Psychopharmacol. Clin. Exp..

[18] Kennedy D., Veasey R., Watson A., Dodd F., Jones E., Maggini S., Haskell C. (2010). Effects of high-dose B vitamin complex with vitamin C and minerals on subjective mood and performance in healthy males. Psychopharmacology.

[19] Harris E., Kirk J., Rowsell R., Vitetta L., Sali A., Scholey A.B., Pipingas A. (2011). The effect of multivitamin supplementation on mood and stress in healthy older men. Hum. Psychopharmacol. Clin. Exp..

[20] Macpherson H., Ellis K.A., Sali A., Pipingas A. (2012). Memory improvements in elderly women following 16 weeks treatment with a combined multivitamin, mineral and herbal supplement. Psychopharmacology.

[21] Herrmann M.H., Schorr R., Obeid J., Scharhag A., Urhausen W., Herrmann W. (2003). Homocysteine increases during endurance exercise. Clin. Chem. Lab. Med..

[22] Herrmann M., Wilkinson J., Schorr H., Obeid R., Georg T., Urhausen A., Scharhag J., Kindermann W., Herrmann W. (2003). Comparison of the influence of volume-oriented training and high-intensity interval training on serum homocysteine and its cofactors in young, healthy swimmers. Clin. Chem. Lab. Med..

[23] Nygård O., Vollset S.E., Refsum H., Stensvold I., Tverdal A., Nordrehaug J.E., Kvåle G. (1995). Total plasma homocysteine and cardiovascular risk profile: The Hordaland Homocysteine Study. JAMA.

[24] Haskell C., Kennedy D., Wesnes K., Milne A., Scholey A. (2007). A double-blind, placebo-controlled, multi-dose evaluation of the acute behavioural effects of guarana’ in humans. J. Psychopharmacol..

[25] Kennedy D.O., Haskell C.F., Wesnes K.A., Scholey A.B. (2004). Improved cognitive performance in human volunteers following administration of guarana (*Paullinia*
*cupana*) extract: Comparison and interaction with *Panax ginseng*. Pharmacol. Biochem. Behav..

[26] Nehlig A. (2010). Is caffeine a cognitive enhancer?. J. Alzheimer’s Dis..

[27] Burke L.M. (2008). Caffeine and sports performance. Appl. Physiol. Nutr. Metab..

[28] Bempong D.K., Houghton P.J. (1992). Dissolution and absorption of caffeine from guaraná. J. Pharm. Pharmacol..

[29] Liguori A., Hughes J.R., Grass J.A. (1997). Absorption and subjective effects of caffeine from coffee, cola and capsules. Pharmacol. Biochem. Behav..

[30] Weckerle C.S., Stutz M.A., Baumann T.W. (2003). Purine alkaloids in *Paullinia*. Phytochemistry.

[31] Espinola E., Dias R., Mattei R., Carlini E. (1997). Pharmacological activity of Guarana (*Paullinia cupana*) in laboratory animals. J. Ethnopharmacol..

[32] Haskell C.F., Kennedy D.O., Milne A.L., Wesnes K.A., Scholey A.B. (2006). The acute behavioural effects of guaraná. Appetite.

[33] Hursel R., Viechtbauer W., Dulloo A.G., Tremblay A., Tappy L., Rumpler W., Westerterp-Plantenga M.S. (2011). The effects of catechin rich teas and caffeine on energy expenditure and fat oxidation: A meta-analysis. Obes. Rev..

[34] Li Y., Wang C., Zhu K., Feng R.N., Sun C.H. (2011). Effects of multivitamin and mineral supplementation on adiposity, energy expenditure and lipid profiles in obese Chinese women. Int. J. Obes..

[35] Kennedy D.O., Stevenson E.J., Jackson P.A., Wishart K., Bieri G., Barella L., Khan J., Forster J., Haskell-Ramsay C.F. The effects of differing cognitive task demands on whole body metabolism and cerebral blood-flow: Modulation by multi-vitamins/minerals and coenzyme Q10.

[36] Scholey A., Bauer I., Neale C., Savage K., Camfield D., White D., Maggini S., Pipingas A., Stough C., Hughes M. (2013). Acute Effects of Different Multivitamin Mineral Preparations with and without Guaraná on Mood, Cognitive Performance and Functional Brain Activation. Nutrients.

[37] Kennedy D.O., Haskell C.F., Robertson B., Reay J., Brewster-Maund C., Luedemann J., Maggini S., Ruf M., Zangara A., Scholey A.B. (2008). Improved cognitive performance and mental fatigue following a multi-vitamin and mineral supplement with added guaraná (*Paullinia cupana*). Appetite.

[38] (2008). Council Directive 2008/108/EC of 15 January 2008. Amending regulation (EC) No 1925/2006 on the addition of vitamins and minerals and of certain other substances to foods. Off. J. Eur. Union.

[39] Institute of Medicine (IOM) (1997). Reference Intakes for Dietary Reference Intakes for Calcium, Phosphorus, Magnesium, Vitamin D, and Fluoride.

[40] Institute of Medicine (IOM) (1998). Reference Intakes for Thiamin, Riboflavin, Niacin, Vitamin B6, Folate, Vitamin B12, Pantothenic Acid, Biotin, and Choline.

[41] Institute of Medicine (IOM) (2000). Dietary Reference Intakes for Vitamin C, Vitamin E, Selenium and Carotenoids.

[42] Institute of Medicine (IOM) (2001). Reference Intakes for Vitamin A, K, Arsenic, Boron, Chromium, Copper, Iodine, Iron, Manganese, Molybdenum, Nickel, Silicon, Vanadium, and Zinc.

[43] Kennedy D.O., Haskell C.F. (2011). Cerebral blood flow and behavioural effects of caffeine in habitual and non-habitual consumers of caffeine: A near infrared spectroscopy study. Biol. Psychol..

[44] Stonehouse W., Conlon C.A., Podd J., Hill S.R., Minihane A.M., Haskell C., Kennedy D. (2013). DHA supplementation improved both memory and reaction time in healthy young adults: A randomized controlled trial. Am. J. Clin. Nutr..

[45] Asamoah S., Siegler J., Chang D., Scholey A., Yeung A., Cheema B.S. (2013). Effect of Aerobic Training on Cognitive Function and Arterial Stiffness in Sedentary Young Adults: A Pilot Randomized Controlled Trial. Physiol. J..

[46] Bond A., Lader M. (1974). The use of analogue scales in rating subjective feelings. Br. J. Med. Psychol..

[47] Ahearn E.P. (1997). The use of visual analog scales in mood disorders: A critical review. J. Psychiatr. Res..

[48] Svebak S., Murgatroyd S. (1985). Metamotivational dominance: A multimethod validation of reversal theory constructs. J. Personal. Soc. Psychol..

[49] Hardy C.J., Rejeski W.J. (1989). Not what, but how one feels: The measurement of affect during exercise. J. Sport Exerc. Psychol..

[50] Borg G.A. (1973). Perceived exertion: A note on “history” and methods. Med. Sci. Sports.

[51] Jeukendrup A., Wallis G. (2005). Measurement of substrate oxidation during exercise by means of gas exchange measurements. Int. J. Sports Med..

[52] Williams C., Nute M.G., Broadbank L., Vinall S. (1990). Influence of fluid intake on endurance running performance. A comparison between water, glucose and fructose solutions. Eur. J. Appl. Physiol. Occup. Physiol..

[53] Gonzalez J.T., Stevenson E.J. (2012). New perspectives on nutritional interventions to augment lipid utilisation during exercise. Br. J. Nutr..

[54] Loy B.D., O’Connor P.J., Dishman R.K. (2013). The effect of a single bout of exercise on energy and fatigue states: A systematic review and meta-analysis. Fatigue Biomed. Health Behav..

[55] Moore R.D., Romine M.W., O’Connor P.J., Tomporowski P.D. (2012). The influence of exercise-induced fatigue on cognitive function. J. Sports Sci..

[56] Tomporowski P.D. (2003). Effects of acute bouts of exercise on cognition. Acta Psychol..

[57] Fredholm B.B., Bättig K., Holmén J., Nehlig A., Zvartau E.E. (1999). Actions of caffeine in the brain with special reference to factors that contribute to its widespread use. Pharmacol. Rev..

[58] Doherty M., Smith P.M. (2005). Effects of caffeine ingestion on rating of perceived exertion during and after exercise: A meta-analysis. Scand. J. Med. Sci. Sports.

[59] Bishop D. (2010). Dietary supplements and team-sport performance. Sports Med..

[60] Supinski G.S., Levin S., Kelsen S.G. (1986). Caffeine effect on respiratory muscle endurance and sense of effort during loaded breathing. J. Appl. Physiol. Respir. Environ. Exerc. Physiol..

[61] D’Urzo R., Jhirad H., Jenne M.A., Avendano I., Rubinstein M., D’Costa R., Goldstein S. (1990). Effect of caffeine on ventilatory responses to hypercapnia, hypoxia, and exercise in humans. J. Appl. Physiol..

[62] Graham T.E. (2001). Caffeine and exercise. Sports Med..

[63] Scholey A., Kennedy D., Wesnes K. (2005). The psychopharmacology of herbal extracts: Issues and challenges. Psychopharmacology.

[64] Kennedy D.O., Veasey R.C., Watson A.W., Dodd F.L., Jones E.K., Tiplady B., Haskell C.F. (2011). Vitamins and psychological functioning: A mobile phone assessment of the effects of a B vitamin complex, vitamin C and minerals on cognitive performance and subjective mood and energy. Hum. Psychopharmacol. Clin. Exp..

[65] Spriet L., MacLean D., Dyck D., Hultman E., Cederblad G., Graham T. (1992). Caffeine ingestion and muscle metabolism during prolonged exercise in humans. Am. J. Physiol..

[66] Hawley J.A., Dennis S.C., Lindsay F.H., Noakes T.D. (1995). Nutritional practices of athletes: Are they sub-optimal?. J. Sports Sci..

