# Supplementary material for: The Effects of Supplementation with a Vitamin and Mineral Complex with Guaraná Prior to Fasted Exercise on Affect, Exertion, Cognitive Performance, and Substrate Metabolism: A Randomized Controlled Trial
Source: Nutrients. 2015 Jul 27;7(8):6109–27. doi: 10.3390/nu7085272 (PMC4555111; doi:10.3390/nu7085272)
Supplement: Supplementary File 1 [file nutrients-07-05272-s001.docx]

**Supplementary Materials**

**Table S1.** Baseline and change from baseline scores for each cognitive measure for each treatment condition.

| **Task** | ***n*** | **Condition** | **COMPASS Tasks (Change from Baseline)** | | | | |
| --- | --- | --- | --- | --- | --- | --- | --- |
|  |  |  | **Baseline Score** | **Pre-Exercise** | **Post-Exercise** | **80 min Post-Exercise** | **Overall** |
| Choice Reaction Time Accuracy (%) | 39 | MVM + G | 97.0 ± 2.7 | 0.9 ± 3.0 | 0.9 ± 2.3 | 0.5 ± 3.0 | 0.8 ± 2.8 |
|  |  | Placebo | 97.2 ± 2.2 | 0.8 ± 2.4 | 0.7 ± 2.7 | −0.5 ± 3.3 | 0.3 ± 2.8 |
| Choice Reaction Time (ms) | 39 | MVM + G | 376.9 ± 47.8 | 1.7 ± 33.6 | −3.5 ± 35.6 | 0.5 ± 38.1 | −0.4 ± 35.7 |
|  |  | Placebo | 370.8 ± 43.0 | 13.9 ± 29.1 | 7.3 ± 30.2 | 9.2 ± 25.9 | 10.1 ± 28.4 |
| RVIP Accuracy (%) | 39 | MVM + G | 53.3 ± 19.1 | 1.3 ± 12.4 | 4.2 ± 11.6 | 4.0 ± 11.3 | 3.2 ± 11.8 |
|  |  | Placebo | 52.3 ± 19.3 | 2.7 ± 8.0 | 2.8 ± 11.9 | 0.0 ± 15.1 | 1.8 ± 11.6 |
| RVIP Reaction Time (ms) | 39 | MVM + G | 491.8 ± 55.4 | −5.8 ± 38.6 | −24.6 ± 46.3 | −20.8 ± 38.9 | −17.1 ± 41.3 |
|  |  | Placebo | 494.7 ± 50.8 | −14.6 ± 36.3 | −19.9 ± 41.2 | −15.0 ± 45.5 | −16.5 ± 41.0 |
| RVIP False Alarms | 39 | MVM + G | 0.9 ± 1.2 | −0.1 ± 1.6 | 0.1 ± 1.2 | 0.2 ± 1.6 | 0.1 ± 1.4 |
|  |  | Placebo | 1.2 ± 1.4 | −0.2 ± 1.6 | −0.6 ± 1.2 | −0.5 ± 1.5 | −0.4 ± 1.4 |
| Numeric Working Memory Accuracy (%) | 36 | MVM + G | 96.0 ± 2.9 | 0.9 ± 2.8 | 0.5 ± 3.1 | 1.0 ± 3.0 | 0.8 ± 2.9 |
|  |  | Placebo | 96.9 ± 2.5 | −1.8 ± 3.6 | −1.5 ± 3.4 | −0.9 ± 3.0 | −1.4 ± 3.3 |
| Numeric Working Memory Reaction Time (ms) | 36 | MVM + G | 721.9 ± 148.9 | −35.8 ± 86.5 | −46.3 ± 99.1 | −72.8 ± 111.2 | −51.6 ± 98.9 |
|  |  | Placebo | 697.6 ± 104.1 | −29.3 ± 70.9 | −30.2 ± 83.5 | −35.2 ± 80.9 | −31.6 ± 78.4 |
| Picture Recognition Accuracy (%) | 38 | MVM + G | 91.4 ± 7.3 | −1.8 ± 6.7 | −1.7 ± 5.9 | −2.0 ± 7.0 | −1.8 ± 6.5 |
|  |  | Placebo | 91.2 ± 8.8 | −2.4 ± 7.2 | −1.1 ± 6.8 | −1.9 ± 7.3 | −1.8 ± 7.1 |
| Picture Recognition Reaction Time (ms) | 38 | MVM + G | 831.5 ± 179.6 | −37.7 ± 127.3 | −59.4 ± 116.1 | −80.4 ± 152.1 | −59.2 ± 131.8 |
|  |  | Placebo | 784.9 ± 103.9 | 5.8 ± 102.3 | −17.7 ± 93.2 | −23.8 ± 105.3 | −11.9 ± 100.3 |
| Word Recognition Accuracy (%) | 38 | MVM + G | 83.2 ± 8.1 | −3.2 ± 8.8 | −2.2 ± 7.7 | −3.9 ± 8.0 | −3.1 ± 8.1 |
|  |  | Placebo | 82.0 ± 8.6 | −2.5 ± 10.9 | −2.1 ± 9.8 | −4.9 ± 8.3 | −3.2 ± 9.7 |
| Word Recognition Reaction Time (ms) | 38 | MVM + G | 850.8 ± 194.3 | −21.0 ± 82.1 | −68.8 ± 134.6 | −85.5 ± 148.7 | −58.4 ± 121.8 |
|  |  | Placebo | 856.7 ± 195.1 | −49.1 ± 135.2 | −87.6 ± 149.5 | −80.5 ± 150.9 | −72.4 ± 145.2 |
| Immediate Word Recall Correct | 39 | MVM + G | 7.9 ± 2.2 | −1.2 ± 2.4 | −0.7 ± 1.8 | −0.8 ± 1.9 | −0.9 ± 2.0 |
|  |  | Placebo | 7.3 ± 2.1 | −0.1 ± 2.1 | 0.1 ± 2.1 | −0.5 ± 2.0 | −0.1 ± 2.1 |

**Table S1.** *Cont.*

| **Task** | ***n*** | **Condition** | **COMPASS Tasks (Change from Baseline)** | | | | |
| --- | --- | --- | --- | --- | --- | --- | --- |
|  |  |  | **Baseline Score** | **Pre-Exercise** | **Post-Exercise** | **80 min Post-Exercise** | **Overall** |
| Immediate Word Recall Errors | 39 | MVM + G | 0.5 ± 0.8 | 0.1 ± 1.0 | −0.1 ± 1.2 | −0.2 ± 0.8 | −0.1 ± 1.0 |
|  |  | Placebo | 0.6 ± 1.2 | −0.1 ± 1.3 | −0.2 ± 1.0 | −0.2 ± 1.1 | −0.2 ± 1.1 |
| Delayed Word Recall Correct | 39 | MVM + G | 5.9 ± 2.5 | −2.1 ± 2.4 | −1.4 ± 2.6 | −2.0 ± 2.2 | −1.8 ± 2.4 |
|  |  | Placebo | 5.3 ± 2.3 | −1.2 ± 2.4 | −0.3 ± 1.8 | −1.9 ± 2.3 | −1.1 ± 2.2 |
| Delayed Word Recall Errors | 39 | MVM + G | 0.7 ± 1.3 | −0.1 ± 1.3 | −0.2 ± 1.1 | 0.1 ± 1.5 | −0.1 ± 1.3 |
|  |  | Placebo | 0.7 ± 1.3 | −0.2 ± 1.1 | −0.3 ± 1.2 | −0.2 ± 1.3 | −0.2 ± 1.2 |

COMPASS: Computerized Mental Performance Assessment System, University of Northumbria at Newcastle; RVIP, rapid visual information processing; MVG + G:
a vitamin and mineral complex with guaraná.

**Table S2.** Baseline and change from baseline ratings for each mood and physical state measure for each treatment condition.

| **Measure** | ***n*** | **Condition** | **Mood Ratings (Change from Baseline)** | | | | |
| --- | --- | --- | --- | --- | --- | --- | --- |
|  |  |  | **Baseline Score** | **Pre-Exercise** | **Post-Exercise** | **80 min Post-Exercise** | **Overall** |
| Alertness | 40 | MVM + G | 54.6 ± 11.7 | 7.7 ± 8.9 | 11.8 ± 11.0 | 6.3 ± 10.6 | 8.6 ± 30.5 |
|  |  | Placebo | 55.1 ± 12.6 | 7.2 ± 9.1 | 10.4 ± 9.6 | 5.9 ± 10.5 | 7.8 ± 29.2 |
| Contentment | 40 | MVM + G | 62.6 ± 9.6 | 2.8 ± 5.0 | 2.8 ± 8.7 | 4.8 ± 8.5 | 3.5 ± 22.2 |
|  |  | Placebo | 63.6 ± 9.7 | 2.9 ± 5.5 | 2.2 ± 6.7 | 3.4 ± 8.1 | 2.8 ± 20.3 |
| Calmness | 40 | MVM + G | 63.4 ± 8.1 | −6.0 ± 8.1 | −13.8 ± 14.4 | 2.9 ± 11.3 | −5.6 ± 33.8 |
|  |  | Placebo | 62.2 ± 7.8 | −4.4 ± 8.6 | −12.5 ± 12.7 | 2.4 ± 11.9 | −4.8 ± 33.3 |
| Concentration | 40 | MVM + G | 53.5 ± 12.3 | 8.2 ± 9.3 | 7.9 ± 14.4 | 3.7 ± 13.2 | 6.6 ± 36.9 |
|  |  | Placebo | 54.6 ± 13.8 | 6.2 ± 10.6 | 5.5 ± 13.6 | 4.2 ± 14.2 | 5.3 ± 38.5 |
| Physical Stamina | 40 | MVM + G | 53.1 ± 12.6 | 7.1 ± 9.1 | 7.4 ± 12.6 | 3.7 ± 13.0 | 6.0 ± 34.8 |
|  |  | Placebo | 53.8 ± 14.5 | 5.1 ± 10.6 | 5.5 ± 12.0 | 4.5 ± 14.5 | 5.0 ± 37.1 |
| Mental Stamina | 40 | MVM + G | 57.5 ± 15.3 | 8.4 ± 5.7 | 6.2 ± 14.3 | 3.7 ± 14.9 | 6.1 ± 34.8 |
|  |  | Placebo | 57.9 ± 15.7 | 4.5 ± 8.4 | 3.8 ± 14.4 | 3.8 ± 11.1 | 4.0 ± 34.0 |

MVG + G: a vitamin and mineral complex with guaraná.**Table S3.** Absolute ratings for each mood and physical state measure for each treatment condition.

| **Measure** | ***n*** | **Condition** | **Pre-Exercise** | **During Exercise** | | | **Post-Exercise** | | **Overall** |
| --- | --- | --- | --- | --- | --- | --- | --- | --- | --- |
|  |  |  |  | **10 min** | **20 min** | **30 min** | **50 min** | **80 min** |  |
| Felt Arousal Scale | 39 | MVM + G | 2.8 ± 1.0 | 3.2 ± 1.1 | 3.4 ± 1.0 | 3.4 ± 1.1 | 2.4 ± 1.0 | 2.5 ± 0.9 | 3.0 ± 1.0 |
|  |  | Placebo | 2.9 ± 1.1 | 3.2 ± 1.0 | 3.3 ± 1.1 | 3.4 ± 1.0 | 2.5 ± 0.9 | 2.4 ± 0.9 | 2.9 ± 1.0 |
| Feeling Scale | 39 | MVM + G | 2.7 ± 1.2 | 2.7 ± 1.2 | 2.5 ± 1.4 | 2.4 ± 1.5 | 2.8 ± 1.1 | 2.7 ± 1.3 | 2.6 ± 1.3 |
|  |  | Placebo | 2.7 ± 1.2 | 2.5 ± 1.1 | 2.3 ± 1.3 | 2.3 ± 1.4 | 2.8 ± 1.1 | 2.8 ± 1.2 | 2.6 ± 1.2 |
| Rating of Perceived Exertion | 40 | MVM + G |  | 10.5 ± 1.6 | 11.4 ± 1.8 | 11.9 ± 1.9 |  |  | 11.3 ± 1.8 * |
|  |  | Placebo |  | 10.7 ± 1.6 | 11.7 ± 1.7 | 12.2 ± 2.0 |  |  | 11.5 ± 1.8 |

MVG + G: a vitamin and mineral complex with guaraná; * Significantly different from placebo (*p* < 0.05).

**Table S4.** Absolute ratings for fat and carbohydrate oxidation during exercise for each treatment condition.

| **Measure** | ***n*** | **Condition** | **During Exercise** | | | **Overall** |
| --- | --- | --- | --- | --- | --- | --- |
|  |  |  | **9–10 min** | **19–20 min** | **29–30 min** |  |
| Fat Oxidation (g/min) | 35 | MVM + G | 0.24 ± 0.16 | 0.37 ± 0.17 | 0.46 ± 0.18 | 0.36 ± 0.16 |
|  |  | Placebo | 0.25 ± 0.15 | 0.39 ± 0.21 | 0.47 ± 0.17 | 0.37 ± 0.17 |
| Carbohydrate Oxidation (g/min) | 35 | MVM + G | 9.88 ± 1.50 | 9.36 ± 1.60 | 8.99 ± 1.57 | 9.41 ± 1.50 |
|  |  | Placebo | 9.80 ± 1.44 | 9.12 ± 1.70 | 8.83 ± 1.61 | 9.25 ± 1.53 |

MVG + G: a vitamin and mineral complex with guaraná.

Significant Effects of Time

Mean scores and ratings are reported with the Standard Deviation (SD). Effect sizes for significant results are reported using Cohen’s D (*d*).

Choice Reaction Time (CRT)

A significant effect of time was found for CRT accuracy (F(2,76) = 4.73, *p* = 0.012), with better performance seen pre- and immediately post-exercise compared to 80 min post-exercise (*p* = 0.18, *d* = 0.28 and *p* = 0.19, *d* = 0.28 respectively).

Rapid Visual Information Processing (RVIP)

A significant effect of time was found for RVIP correct RT (F(2,76) = 5.48, *p* = 0.006), with faster reaction time (RT) seen immediately post-exercise compared to pre-exercise (*p* = 0.014, *d* = 0.30).

Delayed Word Recall

A significant effect of time was found for Delayed Word Recall correct responses (F(2,76) = 8.79,
*p* < 0.0001), with better performance seen immediately post-exercise compared to pre-exercise and
80 min post-exercise (*p* = 0.012, *d* = 0.35 and *p* = 0.001, *d* = 0.49 respectively).

Word Recognition

A significant effect of time was found for Word Recognition RT (F(2,76) = 6.10, *p* = 0.004), with faster RT seen pre-exercise compared to immediately post-exercise and 80 min post-exercise (*p* = 0.023, *d* = 0.34 and *p* = 0.013, *d* = 0.37 respectively).

Picture Recognition

A significant effect of time was found for Picture Recognition reaction time (F(2,76) = 7.59, *p* = 0.001), with faster RT seen at 80 min exercise compared to pre-exercise (*p* = 0.002, *d* = 0.25).

Alertness

A significant effect of time was found for ratings of alertness (F(2,78) = 8.17, *p* = 0.002), which were higher immediately post-exercise compared to pre-exercise (*p* = 0.020, *d* = 0.40) but lower
80 min post-exercise compared to immediately post-exercise (*p* = 0.001, *d* = 0.48).

Calmness

A significant effect of time was found for ratings of calmness (F(2,78) = 49.37, *p* < 0.0001). Ratings were significantly lower immediately post-exercise compared to pre-exercise (*p* < 0.0001,
*d* = 0.71) but higher at 80 min post-exercise compared to pre−exercise (*p* < 0.0001, *d* = 0.77) and immediately post-exercise (*p* < 0.0001, *d* = 1.25).

Felt Arousal Scale (FAS)

A significant effect of time was found for ratings of arousal (F(5,195) = 29.28, *p* < 0.0001). Arousal ratings increased at every time point during exercise compared to pre-exercise (10 min, *d* = 32; 20 min, *d* = 0.46; 30 min, *d* = 0.51; all *p* < 0.0001), but fell significantly by 50 min post exercise (*p* < 0.0001,
*d* = 0.90).

Rating of Perceived Exertion (RPE)

There was a significant effect of time for RPE (F(2,78) = 4.90, *p* = 0.033), which significantly increased at each time point during the exercise period 10–20 min, *d* = 0.60; 20–30 min, *d* = 0.24;
10–30 min, *d* = 0.81; all *p* < 0.0001).

Respiratory Exchange Rate (RER)

There was a significant effect of time for RER (F(2,66) = 138.82, *p* < 0.0001), which significantly decreased at each time point during the exercise period (9–19 min, *d* = 0.91; 19–29 min, *d* = 0.59;
9–29 min, *d* = 1.65; all *p* < 0.0001).

Energy Expenditure (EE)

There was a significant effect of time for EE (F(2,66) = 11.70, *p* < 0.0001) which was significantly higher during minute 29 than during minutes 9 (*p* < 0.0001, *d* = 0.19) and 19 (*p* = 0.002, *d* = 0.10) of the exercise period.

Fat Oxidation

There was a significant effect of time for fat oxidation (F(2,68) = 164.43, *p* < 0.0001), which significantly increased at each time point during the exercise period (9–19 min, *d* = 0.87; 19–29 min,
*d* = 0.56; 9–29 min, *d* = 1.54; all *p* < 0.0001).

Carbohydrate (CHO) Oxidation

There was a significant effect of time for CHO oxidation (F(2,68) = 60.43, *p* < 0.0001), which significantly decreased at each time point during the exercise period (9–19 min, *d* = 0.39; 19–29 min,
*d* = 0.20; 9–29 min, *d* = 0.61; all *p* < 0.0001).

© 2015 by the authors; licensee MDPI, Basel, Switzerland. This article is an open access article distributed under the terms and conditions of the Creative Commons Attribution license (http://creativecommons.org/licenses/by/4.0/).
